# 3D-QSAR and Molecular Docking Studies on the *TcPMCA1*-Mediated Detoxification of Scopoletin and Coumarin Derivatives

**DOI:** 10.3390/ijms18071380

**Published:** 2017-06-27

**Authors:** Qiu-Li Hou, Jin-Xiang Luo, Bing-Chuan Zhang, Gao-Fei Jiang, Wei Ding, Yong-Qiang Zhang

**Affiliations:** Laboratory of Natural Products Pesticides, College of Plant Protection, Southwest University, Chongqing 400715, China; houqiuli2000@126.com (Q.-L.H.); xiangxiangnx@sohu.com (J.-X.L.); zhbichting@163.com (B.-C.Z.); Gaofei.Jiang@toulouse.inra.fr (G.-F.J.); dwing818@163.com (W.D.)

**Keywords:** *Tetranychus cinnabarinus*, plasma membrane Ca^2+^-ATPase, scopoletin, coumarin derivatives, molecular docking, three-dimensional quantitative structure activity relationship (3D-QSAR), interaction mechanism

## Abstract

The carmine spider mite, *Tetranychus cinnabarinus* (Boisduval), is an economically important agricultural pest that is difficult to prevent and control. Scopoletin is a botanical coumarin derivative that targets Ca^2+^-ATPase to exert a strong acaricidal effect on carmine spider mites. In this study, the full-length cDNA sequence of a plasma membrane Ca^2+^-ATPase 1 gene (*TcPMCA1*) was cloned. The sequence contains an open reading frame of 3750 bp and encodes a putative protein of 1249 amino acids. The effects of scopoletin on *TcPMCA1* expression were investigated. *TcPMCA1* was significantly upregulated after it was exposed to 10%, 30%, and 50% of the lethal concentration of scopoletin. Homology modeling, molecular docking, and three-dimensional quantitative structure-activity relationships were then studied to explore the relationship between scopoletin structure and *TcPMCA1*-inhibiting activity of scopoletin and other 30 coumarin derivatives. Results showed that scopoletin inserts into the binding cavity and interacts with amino acid residues at the binding site of the *TcPMCA1* protein through the driving forces of hydrogen bonds. Furthermore, CoMFA (comparative molecular field analysis)- and CoMSIA (comparative molecular similarity index analysis)-derived models showed that the steric and H-bond fields of these compounds exert important influences on the activities of the coumarin compounds.Notably, the C3, C6, and C7 positions in the skeletal structure of the coumarins are the most suitable active sites. This work provides insights into the mechanism underlying the interaction of scopoletin with *TcPMCA1*. The present results can improve the understanding on plasma membrane Ca^2+^-ATPase-mediated (PMCA-mediated) detoxification of scopoletin and coumarin derivatives in *T. cinnabarinus*, as well as provide valuable information for the design of novel PMCA-inhibiting acaricides.

## 1. Introduction

The plasma membrane Ca^2+^-ATPase (PMCA) pumps Ca^2+^ out of the cell to maintain cytosolic Ca^2+^ concentration at a level that is compatible with messenger function. The concentration of nerve membrane Ca^2+^ is normally higher in the cytoplasm than that in the extracellular matrix;furthermore, Ca^2+^ is sequestered by sarco/endoplasmic reticulum Ca^2+^ pumps (SERCA) or by Ca^2+^-binding proteins, or else extruded by Na^+^/Ca^2+^ exchangers or PMCAs [1,2,3]. PMCAs exhibit cell-specific expression patterns and play an essential role in Ca^2+^ homeostasis in various cell types, including sensory neurons [4,5,6,7]. The inhibition of PMCAs in rat and fire salamander cilia by specific drugs, such as vanadate or carboxyeosin, suggests that PMCAs play a predominant role in Ca^2+^ clearance [8,9]. In mammals, four genes encode PMCAs [10]. PMCA isoforms 1 and 4 are ubiquitously expressed and considered as housekeeping isoforms, whereas PMCA isoforms 2 and 3 exhibit limited expression in tissues [4,5,6,7]. Through quantitative analysis, human PMCA1 is shown to be more abundant than PMCA4 at mRNA and protein levels [11]. Numerous methods, such as transient transfection, the use of stable cell lines, and use of the vaccinia viral vector, are used to advance knowledge on the differential properties of these isoforms [12,13,14].

The carmine spider mite, *Tetranychus cinnabarinus* (Boisduval), is a global agricultural pest that parasitizes more than 100 plant species, including beans, cotton, eggplants, tomatoes, and peppers. *T. cinnabarinus* infestations significantly reduce the quality and yield of these crops. These mites are difficult to prevent and control given its high fecundity, short developmental duration, small individual size, limited territory, and high inbreeding rate [15,16]. The control and prevention of *T. cinnabarinus* are currently dependent on chemical insecticides and acaricides, such as spiromesifen, pyridaben, and etoxazole, which introduce a high amount of chemical residues to the environment and induce drug resistance in the target species [17]. Therefore, a novel, environmentally friendly acaricidal compound should be identified and developed to manage these problems.

Scopoletin (7-hydroxy-6-methoxychromen-2-one) is an important coumarin phytoalexin found in many herbs [18]. Scopoletin displays a wide array of pharmacological and biochemical activities [19]. In addition, scopoletin exerts insecticidal, acaridal, antibacterial, and allelopathic activities that are useful in agricultural applications [20,21,22]. A previous study found that scopoletin extracted from *Artemisia annua* L. exhibits strong acaricidal activity against carmine spider mites and inhibits oviposition [22]. Furthermore, many studies on the effects of scopoletin on various protective enzymes in the nervous system of *T. cinnabarinus* indicated that scopoletin inhibits Ca^2+^-ATPase [23]. Thus, scopoletin is has increasingly attracting interest as a potential botanical acaricide because it is more environmentally friendly compared with chemical and physical agents. However, the interaction between Ca^2+^-ATPase and scopoletin in *T. cinnabarinus* remains unclear.

The objective of this study is to investigate the PMCA-meditated detoxification mechanism of scopoletin. Molecular docking and three-dimensional quantitative structure activity relationship (3D-QSAR) analyses were performed to achieve this aim. The full-length cDNA that encodes the PMCA 1 gene (*TcPMCA1*) was obtained from *T. cinnabarinus*. The expression profiles of *TcPMCA1* at the various life stages of carmine spider mites were then reported. The effects of scopoletin on *TcPMCA1* expression during the adult stage of *T. cinnabarinus* were also investigated. The results of the molecular docking and 3D-QSAR studies were used to investigate the mechanism underlying the interaction between scopoletin and *TcPMCA1*, as well as the active site of coumarin compounds. This work provides an insight into the detoxification mechanism of scopoletin at the active site for future studies on the optimized structural design of scopoletin and other coumarin derivatives.

## 2. Results

### 2.1. Cloning and Sequence Analysis

**Figure 1:**
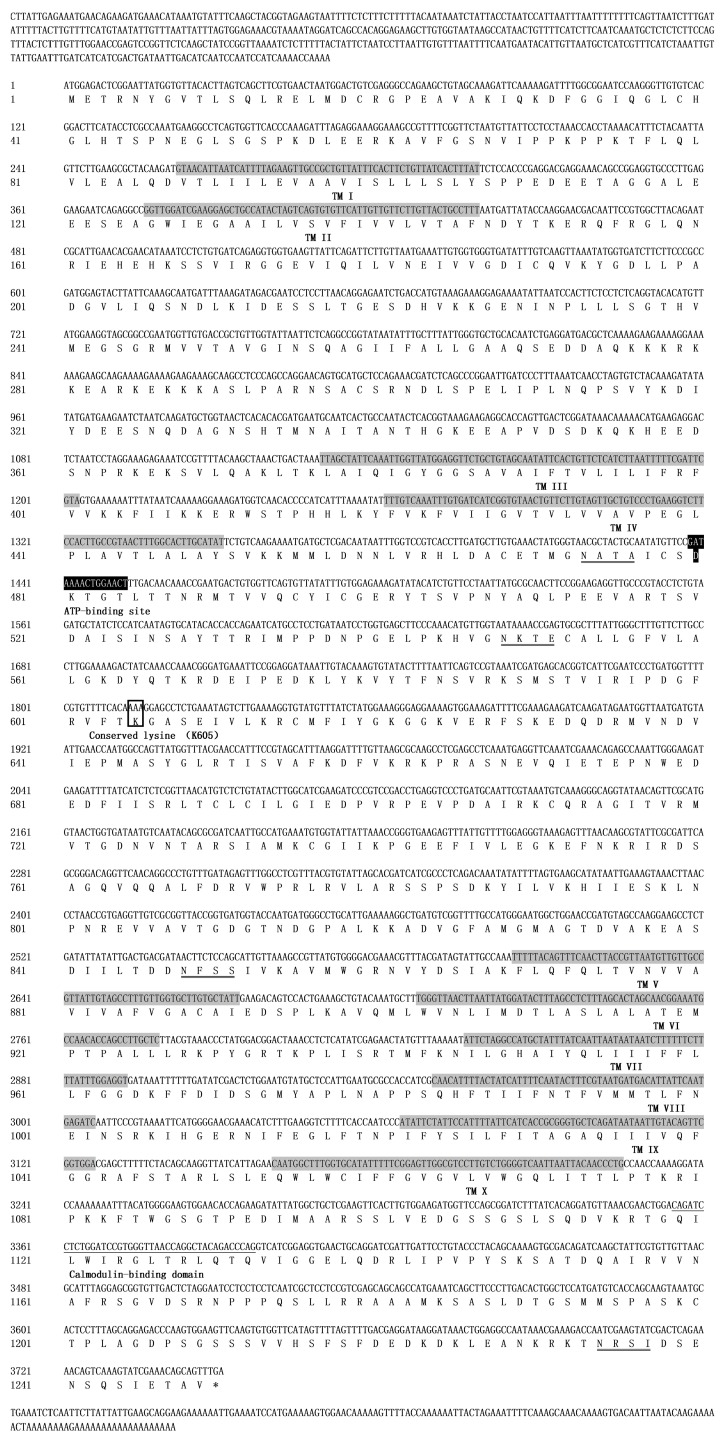
Nucleotide and deduced amino acid sequences of Ca^2+^-ATPase 1 gene (*TcPMCA1*) from the carmine spider mite (*Tetranychus cinnabarinus* (Boisduval)). Nucleotide numbers are provided on the left. The 10 transmembrane (TM) domains, which are denoted as TM I to TM X, are shaded. The ATP (Adenosine Triphosphate)-binding site, together with phosphorylable aspartate (D480), is shaded black, whereas the conserved lysine (K605) is boxed. The calmodulin-binding domain is indicated by a single line and the four *N*-glycosylation sites are indicated by double lines. * represents the termination signal.

The partial cDNA sequence that codes for PMCA1 was identified through the use of transcriptome data and alignment with nucleotide sequences from the genome datasets of *Tetranychus urticae* [24]. The remaining 5′ and 3′ ends were amplified through a RACE (rapid amplification of cDNA ends)/PCR (Polymerase Chain Reaction)-based strategy. The full-length cDNA sequence, which was designated as *TcPMCA1*, was deposited in the GenBank database and with the accession number of KP455490. The full-length cDNA of *TcPMCA1* is 4369 bp in length and contains a 3750-bp open reading frame (ORF), a 456-bp 5′-untranslated region (UTR), and a 163-bp 3′-UTR with a putative polyadenylation signal upstream of the *poly(A)* (Figure 1). The ORF encodes 1249 amino acid residues with a predicted molecular mass of 137.7 kDa and an isoelectric point of 8.10 (Figure 1).

The analysis of the deduced amino acid sequence of *TcPMCA1* revealed the presence of ten membrane-spanning segments (TM), which were denoted as TM I to TM X, as well as four main cytosolic domains located between TM II and TM III, between TM IV and TM V, and at the *N*- and *C*-terminal regions. Some characteristic segments also were predicted. *TcPMCA1* contains an ATP-binding site (from amino acid D480 to T484) and a calmodulin-binding domain (Q1119 to Q1130) (Figure 1).

**Figure 2:**
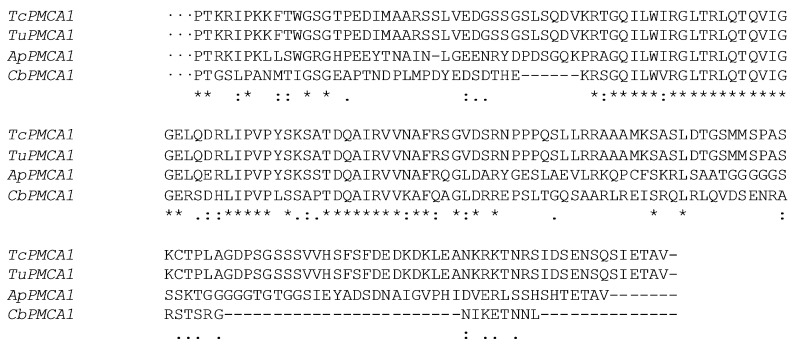
ClustalW alignment of the C-terminal sequence comparison of plasma membrane Ca^2+^-ATPase 1 (*PMCA1*) obtained from different species. Alignment of the sequences of the *PMCAs*, starting after the last (10th) putative membrane-spanning domain and ending at the last residue. Residues that are completely conserved are marked with an asterisk (*); those that are highly conserved are indicated by colon (:); while similar residues are indicated by a dot (.). “-” represents interval. *PMCA1* sequences used in the alignment are as follows: *TcPMCA1*, *Tetranychus cinnabarinus*; *TuPMCA1*, *Tetranychus urticae*; *ApPMCA1*, *Acythosiphon pisum*; and *CbPMCA1*, *Caenorhabditis briggsae*. The *PMCA2* sequences used in the alignment are as follows: *TcPMCA2*, *Tetranychus cinnabarinus*; *TuPMCA2*, *Tetranychus urticae*; *IsPMCA*, *Ixodes scapularis*; and *CbPMCA1*, *Caenorhabditis briggsae*.

The multiple protein alignments of the C-terminal conserved catalytic domains of the PMCAs from Arachnida and insects showed that *TcPMCA1* exhibits 99.7% amino acid sequence identity with *T. urticae* PMCA1. *TcPMCA1* also showed nearly 70% similarity with the PMCA genes of *Ixodes scapularis*, and 60–75% similarity with the PMCA genes of insects and nematodes (Figure 2).

### 2.2. Phylogenetic Analysis

**Figure 3:**
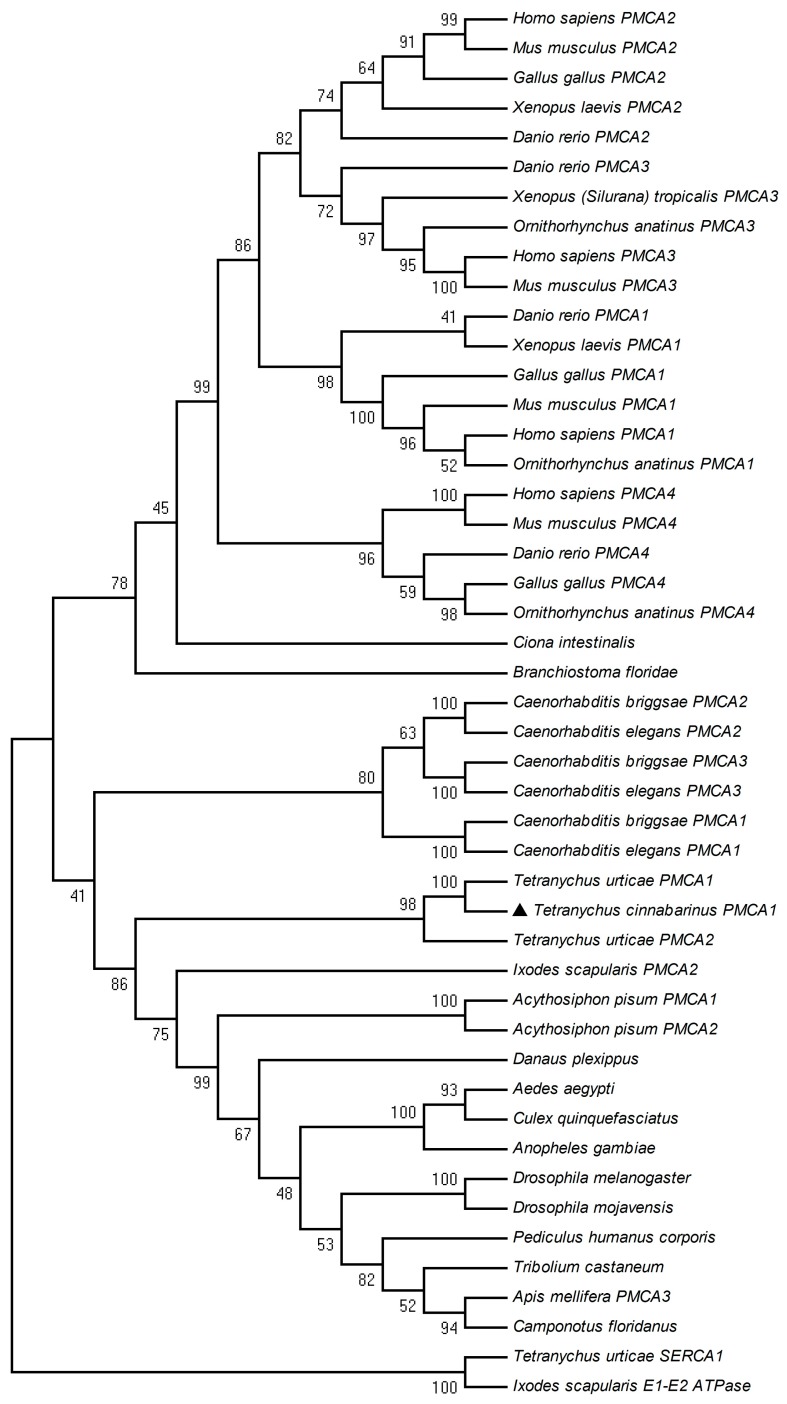
Phylogenetic analysis of *TcPMCA1* obtained from the carmine spider mite (*Tetranychus cinnabarinus* (Boisduval)). The phylogenetic tree was constructed using Molecular Evolutionary Genetics Analysis (MEGA) 5.04 using the neighbor-joining method based on amino acid sequences. *TcPMCA1* was indicated by “▲”. Bootstrap support values derived from 1000 replicates are shown on the branches. Sequence accession numbers are given in Electronic Supplementary Material, Table S1.

A neighbor-joining phylogenetic tree was constructed by comparing the amino acid sequence of *TcPMCA1* with those of PMCA genes from other animal species. Phylogenetic analysis showed that *TcPMCA1* belongs to the cluster of *Ixodes* PMCA. The PMCA genes of *T. cinnabarinus* and *T. urticae* clustered into the PMCA family and apparently share a single clade. These results suggested that the PMCA genes of *T. cinnabarinus* and *T. urticae* are evolutionarily related and share similar physiological functions (Figure 3).

### 2.3. Developmental Expression Patterns

**Figure 4:**
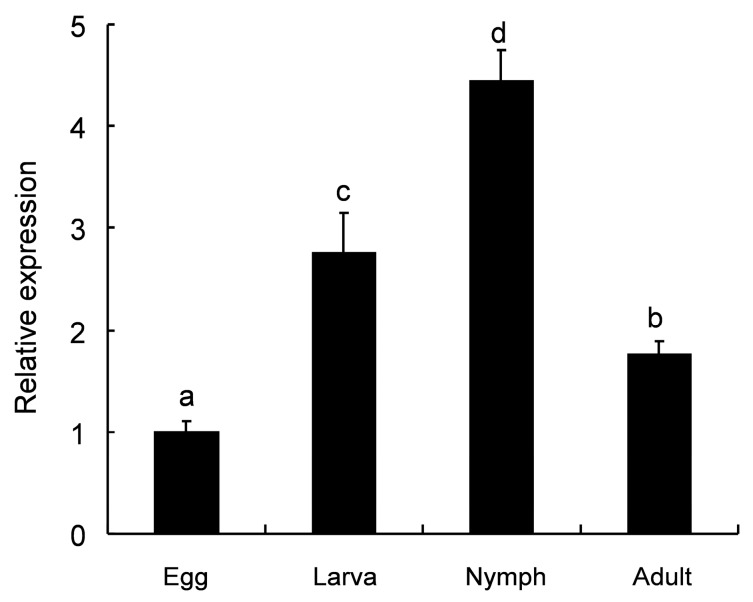
Expression levels of the plasma membrane Ca^2+^-ATPase 1 gene (*TcPMCA1*) at different developmental stages of *Tetranychus cinnabarinus* were evaluated using Real-time Quantitative polymerase chain reaction (RT-qPCR). The egg, larval, nymphal, and adult stages were analyzed. Relative expression was calculated according to the value of the lowest expression level, which was assigned with an arbitrary value of 1. Letters above the bars indicate significant differences among different developmental stages. *RPS18* was used as reference gene. Data were presented as the means (±SE) of three biological replications per developmental stage. Different letters on the error bars indicate significant differences revealed by ANOVA test (*p* < 0.05).

To gain insights into the potential role of *TcPMCA1*, the expression levels of *TcPMCA1* in female individuals at various life stages were quantified through Real-time Quantitative polymerase chain reaction (RT-qPCR). The results showed that *TcPMCA1* mRNA was detected at all developmental stages, including the larval, nymphal, and adult stages. More specifically, the *TcPMCA1* transcript was slightly detectable at the egg stage, was highly expressed at the larval, nymphal, and adult stages, and was the highest at the nymphal stage (Figure 4).

### 2.4. Effects of Scopoletin Exposure on TcPMCA Expression

**Figure 5:**
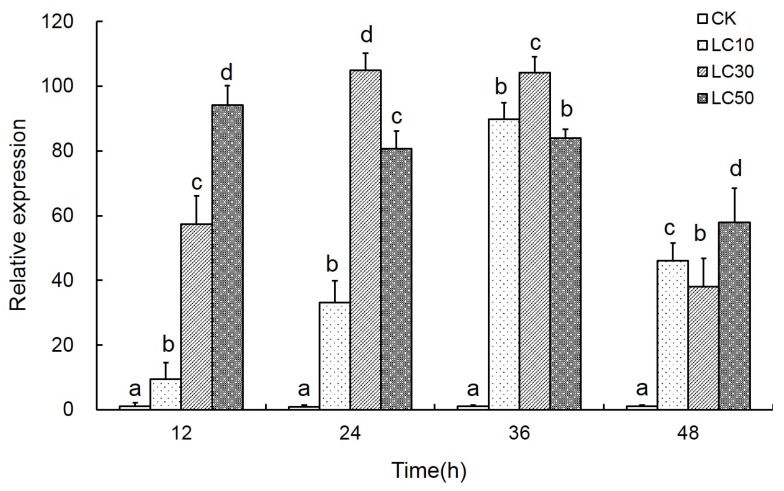
Relative expression levels of the *TcPMCA1* gene in adult female *Tetranychus cinnabarinus* exposed LC_10_ (0.219 mg mL^−1^), LC_30_ (0.581 mg mL^−1^), and LC_50_ (1.142 mg mL^−1^) scopoletin. Expression levels were quantified using qPCR after 12, 24, 36, and 48 h of treatment through leaf-dip bioassay (*n* = 3). Scopoletin was mixed with acetone and Tween-80 (scopoletin: Tween-80 = 3:1; acetone was added until scopoletin dissolved, generally limited within 5%). *T. cinnabarinus* treated with double distilled water containing 0.5% acetone and Tween-80 were used as controls (CK). The mRNA levels in the control and in each treatment were normalized to the expression of the reference gene *RPS18.* The mean expression in each treatment was shown as fold change compared with the mean expression in the control, which was assigned with a basal value of 1. Letters on the error bar indicate significant difference between scopoletin treatment and control (*p* < 0.05).

Scopoletin exposure caused spasms and high mortality among adult *T. cinnabarinus*. The results of induction showed that exposure to scopoletin significantly changed the *TcPMCA1* expression. *TcPMCA1* was significantly upregulated following exposure to low lethal (LC_10_), sublethal (LC_30_), and median lethal (LC_50_) scopoletin concentrations for 12, 24, 36, or 48 h. The relative expression levels of *TcPMCA1* were upregulated by more than 100-fold of that of the control following 24 or 36 h of exposure to scopoletin at LC_30_ dose. However, *TcPMCA1* activation by scopoletin weakened gradually with the extension of time (Figure 5).

### 2.5. Homology Modeling

**Figure 6:**
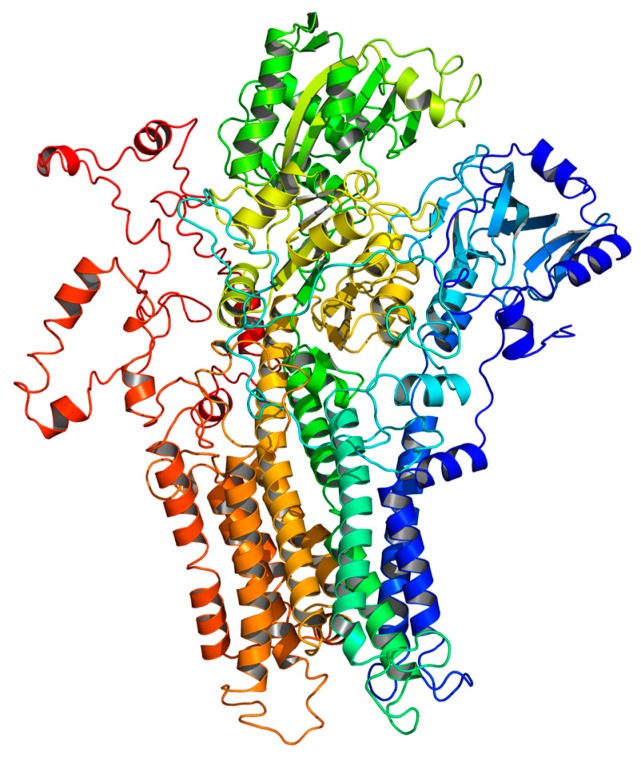
Homology modeling 3D-structure of *TcPMCA1*.

Bell Labs Layered Space-Time (BLAST) analysis revealed that the primary sequence of the target enzyme had a high sequence identity of 73% with the template 3BA6. BLAST analysis guarantees that the model structure is of a high quality. Further energy minimization was performed to remove geometric restraints prior to model construction [25]. The homology modeling of *TcPMCA1* is shown in Figure 6. The 3D structure of this enzyme was further checked by Procheck to evaluate the stereo-chemical quality. Ramachandran plot analysis showed that most residues are present at the most favored regions. In particular, 90.3% of the residues were in the most favored regions, 9.0% residues in the additional allowed regions, giving a total of 99.3%. Other 0.4% residues in the generously allowed regions and 0.4% residues in the disallowed regions. The results of the procheck analysis demonstrated that the 3D-modeling structure exhibits reasonable and reliable stereo-chemical properties and is thus appropriate for subsequent molecular docking study.

### 2.6. Molecular Docking

**Figure 7:**
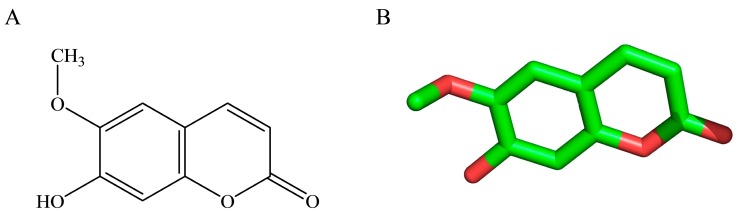
(**A**) Chemical structural formula and (**B**) the cartoon representation of scopoletin. Red regions represent oxygen atoms; green regions represent carbon atoms.

**Figure 8:**
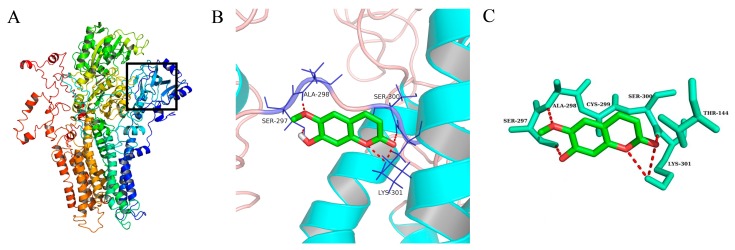
(**A**) Binding pocket of *TcPMCA1* was indicated by the black frame; (**B**) best conformation of scopoletin docked to binding pocket of *TcPMCA1*; (**C**) cartoon representation of residues involved in the binding of scopoletin to *TcPMCA1*. The black box represents the binding cavity. Short, red dashed lines represent hydrogen bonds. Red regions represent oxygen atoms of scopoletin; green regions represent the carbon atoms of scopoletin; the others represent the amino acid residue of the protein.

To comprehend the interaction between the ligand scopoletin and TcPMCA1, molecular docking was performed to investigate the binding mode of scopoletin within the binding pocket of TcPMCA1, and to further understand their structure–activity relationship. The ligand structure of scopoletin is shown in Figure 7. The result showed that scopoletin docked with high affinity to the nucleotide-binding pocket of TcPMCA1 and amino acid residues Ser297 and 300, Thr144, Cys299, Glu83, Gln86, Asp87, and Lys301 surrounded scopoletin. Furthermore, five hydrogen bonds (the red dash lines) formed between the 7-hydroxy with Sre297, 6-methoxy with Ala298, oxygen at position 1 with Lys301, and oxygen at position 2 with Lys301 and Ser300 (Figure 8).

The 30 coumarin derivatives (Table 1) were also subjected to molecular docking calculations. The derivatives all docked with high affinity to the nucleotide-binding domain (NBD). These results appeared promising and encouraged the calculation of molecular docking at the NBD for all compounds. Defined molecular docking (DMD) at the nucleotide-binding pocket revealed that all compounds showed low binding energy values. The lowest binding energy of −6.03 kcal/mol was exhibited by compound 2 (Table 1). Therefore, compound 2 appears to be the most stable compound.

### 2.7. CoMFA and CoMSIA Statistical Result

The same training (24 compounds) and test sets (six compounds) (Table 2) were used to derive models through CoMFA and CoMSIA. The statistical details were summarized in Table 3. The results showed that the optimal CoMFA model provided a leave-one-out *q*^2^ of 0.75 (>0.5) with an optimal number of principal components (ONC) of 7. A correlation coefficient *R*^2^ of 0.993 with a low standard error of the estimate (SEE) of 0.042, and an *F*-statistic value of 383.856 were also obtained. In contribution, the CoMFA steric field and electrostatic field contributed 72.6% and 27.4%, respectively. The best CoMSIA model provided a *q*^2^ of 0.71 with an ONC of 6. An *R*^2^ of 0.975 with a low SEE of 0.080 and an *F* value of 124.834 were obtained. In CoMSIA model, the contributions of the steric, electrostatic, hydrophobic, H-bond donor and acceptor were 14.0%, 33.4%, 23.9%, 19.7%and 9.0%, respectively (Table 3). Based on these field contributions, the steric field is the most important field in the CoMFA model, whereas the electrostatic field is the most important field in the CoMSIA model.

**Figure 9:**
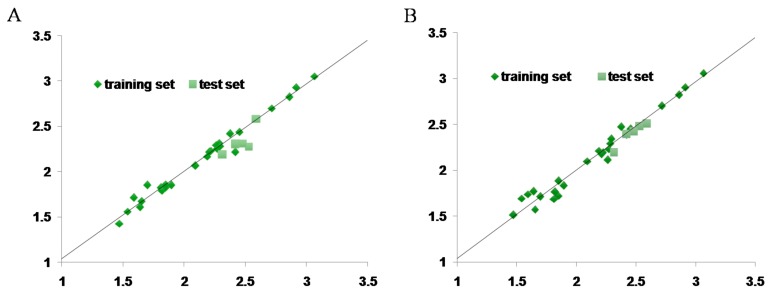
Plots of experimental activity [log (1/LC_50_)] against activity as predicted using CoMFA- (**A**) and CoMSIA-derived (**B**) models.

The test set (six compounds) was used to evaluate the predictive accuracy of the CoMFA and CoMSIA models. Table 4 showed the experimentally determined and predicted activitiesand the training and test sets residual values. The residual values obtained by calculating the difference between the predicted and actual pLC_50_ are below one logarithmic unit for all the compounds (Figure 9). Therefore, the predictive abilities of the optimal CoMFA/CoMSIA models are excellent.

### 2.8. Contour Maps of CoMFA-Derived Models

**Figure 10:**
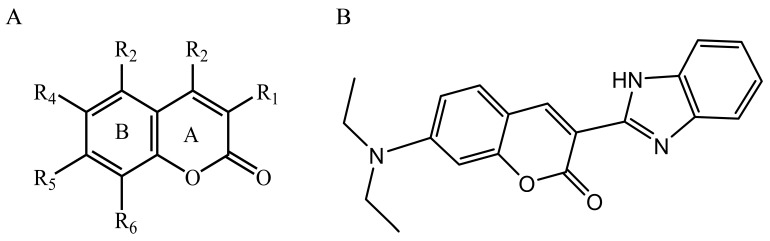
(**A**) Core structure of the test compounds and (**B**) the chemical structure of compound 2.

**Figure 11:**
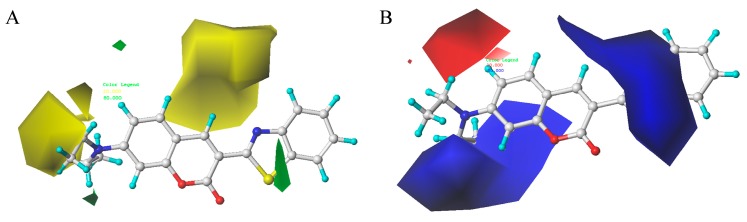
Steric (**A**) and electrostatic (**B**) contour maps obtained using CoMFA-derived models based on molecule 2. Green regions (**A**) indicates regions where the introduction of a bulky group would increase activity. Yellow regions (**A**) indicates regions where the introduction of a bulky group would decrease activity. Red regions (**B**) indicates regions where the introduction of electronegative groups is favored. Blue regions (**B**) indicates regions where the introduction of electropositive groups is favored. The others in Figure A and B represent the compound 2 (Red, oxygen atoms; yellow and blue, nitrogen atom; cyan, hydrogen atom; gray, carbon atoms).

Stdev * Coeff contour maps were plotted on the basis of the optimal CoMFA/CoMSIA-derived models. Core structure of these test compounds were shown in Figure 10A. Compound 2 was employed as the template molecule for the analysis of contour maps (Figure 10B) because of it had the highest acaricidal effect and its lowest binding energy among all compounds. Figure 11 presents the steric and electrostatic contour maps for the optimal CoMFA-derived models. The green and yellow contours in the contour maps indicated default 80% and 20% contribution levels, respectively. From Figure 11A, a medium-sized green contour near the R5-position of ring B indicated that inhibitory activity could be improved with a bulky substituent introduced in this region. Correspondingly, other compounds have bulky substituents at this position. Another green contour occurred around the R1-position of ring A, suggesting that inserting a bulky group into ring A increases inhibitory activity. By contrast, a large yellow contour near the R5-position of ring B implied that the introduction of a bulky group at this position negatively affects inhibitory activity. Another large yellow contour around the R2 and R3 positions suggested that inserting a bulky group in these positions decreases inhibitory activity. Indeed, the inhibitory activities of compounds 1–4 (with a group at R1-or R5-position) are higher than that of compound 23 (with an H atom at this position; Table 2).

Figure 11B showed the electrostatic contour maps obtained from CoMFA-derived models. Red contour indicates electronegative groups are favored; blue contour indicates electropositive groups are favored. These contours depict default contribution levels. A large blue contour near the R5 and R6 positions of ring B suggested that the introduction of electronegative groups in this position will decrease inhibitory activity. Another large blue contour near the R1-position of ring A indicated that the introduction of electropositive groupsenhances inhibitory activity. A large red contour near the R4-positions of ring B suggested that replacing the original groups with electronegative groups at these positions could improve inhibitory activity. For example, the inhibitory activities of compounds 3 (R1 = –NH_2_) and 4 (R1 = –COCH_3_) are greater than that of compound 23 (R1 = –H), and the inhibitory activity of compound 8 (R4 = –NO_2_) is greater than that of compound 23 (R1 = –H) (Table 2).

### 2.9. Contour Maps of CoMSIA-derived Models

**Figure 12:**
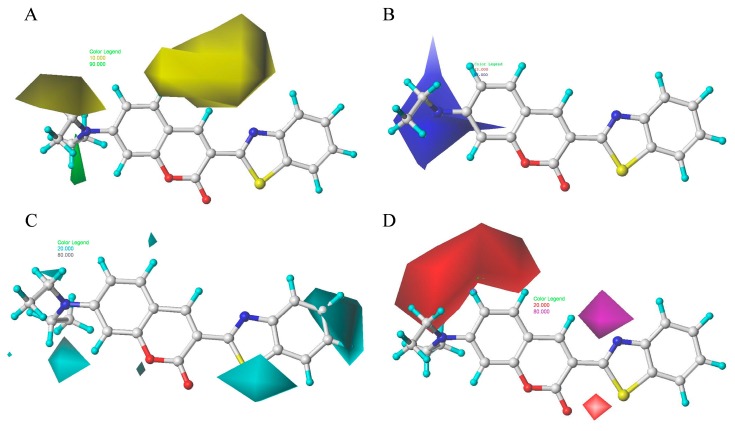
Steric (**A**), electrostatic (**B**), hydrophobic (**C**), and H-bond (**D**) contour maps obtained using CoMSIA-derived models based on molecule 2. Green (**A**) indicates regions where the introduction of a bulky group would increase activity. Yellow (**A**) indicates regions where the introduction of a bulky group would decrease activity. Blue (**B**) indicates regions where the introduction of electropositive groups is favored. Cyan (**C**) indicates regions where the introduction of hydrophobic is favored. Purple (**D**) indicates regions where the introduction of H-bond acceptors is favored. Red (**D**) indicates regions where the introduction of H-bond acceptors is disfavored. The others in Figure **A**–**D** represent the compound 2 (Red, oxygen atoms; yellow and blue, nitrogen atom; cyan, hydrogen atom; gray, carbon atoms).

The steric, electrostatic, hydrophobic, and H-bond contour maps for the optimal CoMSIA-derived models are shown in Figure 12. Figure 12A,B show the steric and electrostatic contour maps, respectively, which were obtained from the optimal CoMSIA model. The CoMSIA steric and electrostatic contour maps are similar to the corresponding CoMFA contour map. Therefore, the preceding discussion also applies to the steric and electrostatic contour maps from the CoMFA model.

Figure 12C shows the hydrophobic contour map of the CoMSIA model is displayed. In the CoMSIA-derived hydrophobic field, a medium-sized cyan contour near the ring B indicated that introducing hydrophilic groups to that position could improve the inhibitory activity of the molecule. Another two yellow contours around the R1-position of ring A suggested that hydrophobic groups preferentially localize at these positions. Figure 12D shows the H-bond contour map for the optimal CoMSIA model. In this figure, the cyan color indicated regions that favor H-bond donors, whereas the red color indicated regions that disfavor H-bond donors. A medium-sized cyan contour occurred at the 2-position on ring A, thus indicating that the inhibitory activity would be improved with an H-bond acceptor group introduced at this position. A large red contour near the 4-position of ring B implied that introducing an H-bond donor group in this position could decrease inhibitory activity.

**Figure 13:**
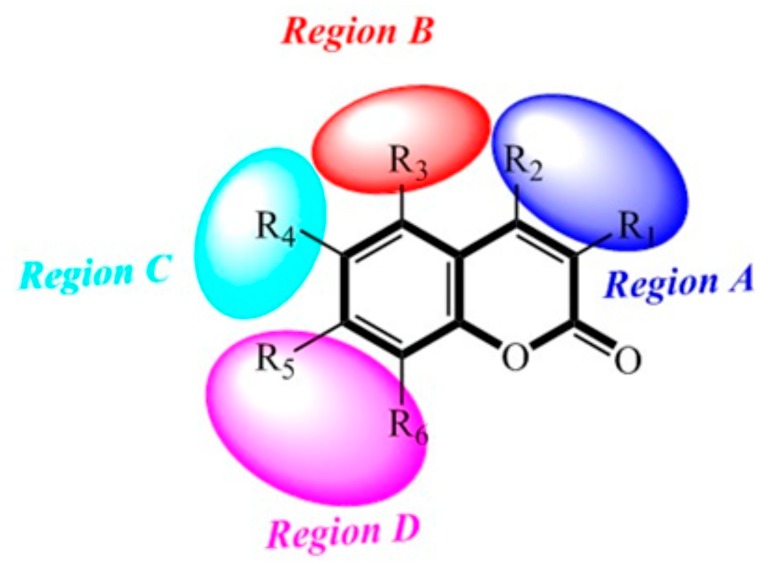
Diagram of structure–activity relationship based on the core structure of the tested compounds. Blue (region A) indicates regions where the introduction of electropositive group, hydrophobic group, or H-accepting groups would increase activity. Red (region B) indicates regions where the introduction of hydrophobic group is favored. Cyan (region C) indicates regions where the introduction of electronegative group is favored. Magenta (region D) indicates regions where the introduction of a bulky group, hydrophobic group, or electropositive group would increase the activity. Dark indicates the core structure of the test compounds.

The detailed analysis of the contour maps obtained using the optimal CoMFA- and CoMSIA-derived models may facilitate the design of a novel selective TcPMCA1 inhibitors. Introducing an electropositive, hydrophobic, or H-accepting group in region A (R1- and R2-positions of ring A) can increase inhibitory activity, and introducing a hydrophobic group in region B (R3-position of ring B) can increase activity. Meanwhile, introducing an electronegative group in region C (R4-position of ring B) is favorable, and introducing a bulky, hydrophobic, or electropositive group in region D (R5- and R6-position of ring B) can increase activity (Figure 13).

## 3. Discussion

Scopoletin is a naturally occurring, low-molecular-weight alloleochemical that is ubiquitous in the plant kingdom. Moreover, scopoletin is present in some foods and plant species used in traditional medicine. Scopoletin extracted from *Artemisia annua* L. exhibits strong activity against the carmine spider mite; in addition, it affects ATPase activity and is possibly a neurotoxin [22].

In the present study, full-length cDNA encoding PMCA1 from *T. cinnabarinus* was characterized and designated as *TcPMCA1*. The predicted amino acid sequences of *TcPMCA1* consists of three major regions: the first intracellular loop region located between transmembrane segments TM II and TM III; the second large intracellular loop region located between TM IV and TM V; which possesses a putative ATP-binding site; the third part extended “tail” found next to TM X. This conformation is consistent with the structure of previously described PMCAs [26,27,28,29]. The putative CaM-binding domain of *TcPMCA1* binds to the C-terminal region downstream of the last transmembrane domain and shares a common pattern with those in vertebrates [30]. Alternative splicing expands the diversity of mRNA transcripts and augments the functions of modulatory genes [31]. Previous efforts to discriminate *TcPMCA1* splice variants failed, this failure was also reported in *Spodoptera littoralis* [32]. By contrast, mammals and *Drosophila melanogaster* possess a large number of splice variants [28].

The expression profiles of *TcPMCA1* in *T. cinnabarinus* were similar to that in *S. littoralis*, which is present at all investigated stages and exhibits maximal expression at the nymphal stage [32]. This expression pattern is correlated to the massive synthesis of TcPMCA1 during the developmental stages, thereby confirming that TcPMCA1 is essential for the functions of *T. cinnabarinus*.

The reported pharmacological effects of scopoletin presuppose some interactions with membrane-bound enzymes, such as Ca^2+^-ATPase, which is vital in nervous signal conduction [33,34,35]. Oliveira [36] reported that in rats, scopoletin inhibits Ca^2+^-ATPase activity by inhibiting the mobilization of intracellular calcium from noradrenaline-sensitive Ca stores. Ca^2+^-ATPase is a major neurotransmitter, and PMCA extrudes Ca^2+^ from the postsynaptic region of the nerve [37]. In insects, PMCA inhibition results in internal Ca^2+^ flow, causing neurotransmitter accumulation [38]. In the present study, the results of scopoletin induction indicated that *TcPMCA1* in *T. cinnabarinus* was significantly upregulated after exposure to scopoletin within 36 h. Scopoletin also increases the expression of both peroxisome proliferator-activated receptor γ2 and adipocyte-specific fatty acid binding protein [39]. Moreover, scopoletin inhibits the expression of cyclooxygenase in a concentration-dependent manner [40]. These results implicated *TcPMCA1* in the detoxification metabolism of scopoletin in *T. cinnabarinus*. The inhibition of Ca^2+^-ATPase activity or increase in PMCA expression possibly indicates the existence of a feedback regulatory mechanism that compensates for enzyme content. The decrease of gene expression at 48 h may related to the organism damage caused by continuous scopoletin exposing. Basing on these results, we surmise that *TcPMCA1* inhibition in *T. cinnabarinus* causes intra- and extracellular calcium ion imbalance and thus blocks the transmission of neural activity, causing the death of mites [41,42].However, the influence of scopoletin on Ca^2+^-ATPase mechanism in the carmine spider mite requires extensive exploration because of the intricacy of PMCA-mediated detoxification.

Scopoletin is also designated as 7-hydroxy-6-methoxy coumarin and is a coumarin derivative. Coumarin is a leading molecule in biopesticides. Given the pesticidal potential of this class of compounds, the toxic effects of coumarin derivatives against mosquito species *Culex quinquefasciatus* and *Aedes aegypti* were evaluated, and the results showed that modifying the 7-OH position remarkably enhances the ovicidal activity of coumarin [43]. The antitermiticidal activity of scopoletin and coumarin derivatives were investigated against *Coptotermes formosanus*, and the results suggested that scopoletin has the highest activity among the tested compounds [44]. To investigate the structure–activity relationship of the methoxy and hydroxy groups at the C-6 and C-7 positions of the coumarin skeleton, 6-alkoxycoumarin derivatives and 7-alkoxycoumarins and related analogs were synthesized. The findings indicated that the presence of alkenyloxy and alkynyloxy groups at the C-6 position, as well as the cyclohexyloxy and aryloxy groups at the C-7 position, are important for the termiticidal and antifeedant activities of coumarin [45,46]. These results revealed that scopoletin actually inserts into the binding cavity and interacts with the active sites of TcPMCA1, suggesting that the microenvironments and conformation of the enzymes change because of these interactions [47]. Furthermore, these results indicated that the C-6 and C-7 positions of scopoletin are important for acaricidal activity.

Molecular docking and the homology modeling of the 3D structure of the target protein were used to identify conformational protein–ligand interaction patterns [48,49]. Pharmacophore have been used to develop 3D-QSAR models over the past the decade [50]. Combined information on protein–ligand interactions from a pharmacophore and accurate binding conformations from molecular docking offers the potential for enhanced prediction accuracy [51]. In the present study, the crystallographic structure of sarco/endoplasmic reticulum Ca^2+^-ATPases (SERCA) was defined in rabbit [52]. The BLAST analysis performed showed that TcPMCA1 shares 73% sequence identity with the SERCA Ca^2+^-ATPase of rabbit, indicating the validity of homologous protein structure [53,54]. The homologous 3D structure of TcPMCA1 allowed the evaluation of the binding energies and docking positions of scopoletin on TcPMCA1 protein. In our docking results, the hydrophobic environment of the active site is favorable for interactions with scopoletin, and the special arrangements at the C6 and C7 sites are assumed to be favorable for the acaricidal activity of scopoletin. Furthermore, the 3D-CoMFA and CoMSIA models indicating that C3, C6, and C7 regions of coumarins appear to be important acaricidal active sites of coumarins. This result is in agreement with the results of the acaricidal activity assay, which showed that coumarins substituted with methoxy at C6 or C7 have significantly better activity than coumarins substituted with other compounds at the same positions. Furthermore, coumarins with C3 substitutions also demonstrated enhanced acaricidal activity. Nakamura [55] previously investigated the structure–activity relationship between 63 natural oxycoumarin derivatives and their effects on the expression of inducible nitric oxide synthase, which showed that the C-5, C-6 and C-7 positions of oxycoumarin derivatives are essential for potent activities. In addition, the discovery and structure–activity relationship of a novel series of coumarin-based tumor necrosis factor α (TNF-α) inhibitors showed that substitution at the C-3 and C-6 position of the coumarin ring system most dramatically influences inhibitory activity against TNF-α [56]. The docking results and the detailed analysis of the contour maps obtained by 3D-CoMFA and CoMSIA-derived models will encourage the design of novel, selective *TcPMCA* inhibitors.

## 4. Materials and Methods

### 4.1. Test Mites

The carmine spider mite culture was collected from cowpea *Vigna unguiculata* (L.) grown in Beibei, Chongqing, China. The mites were maintained on potted cowpea seedlings (30–40 cm tall) in a walk-in insect rearing room at 26 ± 1 °C under 75 to 80% RH and 16L:8D photoperiod. The colony was maintained for more than 12 years without any contact with insecticides/acaricides. The voucher specimens of *T. cinnabarinus* were deposited at the Insect Collection of Southwest University, Chongqing, China.

### 4.2. Leaf-Dip Bioassay

More than 600 leaf discs were prepared to obtain uniform individuals at different developmental stages. Fresh cowpea leaves that had not been exposed to pesticides were washed thoroughly. Leaf discs with 3 cm diameters were placed on a 4 mm water-saturated sponge in a Petri dish (9 cm in diameter) [57]. Approximately 30 adult females were transferred to each leaf disc, allowed to lay eggs, and removed after 12 h. After a batch of uniform eggs had hatched, the offspring was maintained until the progeny had developed into 3- to 5-d-old females [58].

For the leaf-dip bioassay, female adult mites were treated with scopoletin (provided by Southwest University, Beibei, Chongqing, China). The responses of *TcPMCAs* in mites to scopoletin were investigated by exposing the adult female mites to 10% of the lethal concentration (LC_10_), LC_30_, and LC_50_ of scopoletin for 12, 24, 36, and 48 h. The LC_10_ (0.219 mg·mL^−1^), LC_30_ (0.581 mg·mL^−1^), and LC_50_ (1.142 mg·mL^−1^) of *T. cinnabarinus* to scopoletin were determined using leaf-dip bioassays prior to acaricide treatments. Each leaf disc, which contained 30 mites on its surface, was soaked for 5 s in acaricide solutions. For each treatment, more than 500 surviving mites were collected and three biological replicates were performed. A total of 200 mites were dipped in distilled water for 5 s and used as the control. All of the surviving mites were collected and stored at −80 °C for RNA extraction.

### 4.3. RNA Isolation and Reverse Transcription

Total RNA was isolated using RNeasy^®^ Plus Micro Kit (Qiagen, Hilden, Germany), and genomic DNA was removed using a gDNA elimination column in accordance with the manufacturer’s instructions. The quantities of total RNA were assessed at 260 nm using Nanovue UV-Vis spectrophotometer (GE Healthcare, Fairfield, CT, USA). RNA purities were quantified at an absorbance ratio of OD260/280. RNA integrity was evaluated via 1% agarose gel electrophoresis. cDNA was synthesized using total RNA and the rapid amplification of cDNA ends (RACE) method. First-strand cDNA was synthesized from 0.5 µg of RNA in a 10 µL reaction mixture by using PrimeScript^®^ 1st strand cDNA Synthesis Kit (TaKaRa, Dalian, China) and oligo (dT)18 primers. The synthesized samples were then stored at −20 °C.

### 4.4. Sequencing and Phylogenetic Analysis

To obtain the full-length DNA sequences of *TcPMCA* genes, specific primers were designed using Primer 5.0 (Available online: http://www.premierbiosoft.com/) based on the transcript unigene sequences obtained from the transcriptome (Table S2). A set of gene-specific primers and nested primers were designed to amplify the fragments. The rapid amplification of cDNA ends (RACE) methodwas amplified using the SMARTer™ RACE cDNA Amplification Kit (Clontech, Palo Alto, CA, USA). The total PCR volume was 25 µL and contained 2.5 µL of 10× PCR buffer (Mg^2+^ free), 2.0 µL of dNTPs (2.5 mM), 2.0 µL of Mg^2+^ (2.5 mM), 1 µL of cDNA templates, 1 µL of each primer (10 mM), 0.25 µL of rTaq™ polymerase (TaKaRa), and 15.5 µL of ddH_2_O. The PCR program was performed as follows: initial denaturation for 3 min at 94 °C, followed by 34 cycles of 94 °C for 30 s, 55 to 60 °C (depending on gene specific primers) for 30 s, and 72 °C extension for 2 min, and final extension for 10 min at 72 °C. The PCR products were separated by agarose gel electrophoresis andpurified using Gel Extraction Mini Kit (Watson Biotechnologies, Shanghai, China). The purified PCR products were ligated into the pGEM-T vector (Promega, Fitchburg, MA, USA) and then sequenced (Invitrogen Life Technologies, Shanghai, China).

BLAST searching was performed using the NCBI BLAST website (Available online: http://www.ncbi.nlm.nih.gov/Blast.cgi). The molecular weight and isoelectric points of the deduced protein sequences were calculated by ExPASy Proteomics Server (Available online: http://cn.expasy.org/tools/pi_tool.html) [59]. The transmembrane domain positions and protein domain were estimated using Phobius (Available online: http://phobius.sbc.su.se/), Calmodulin Target Database (Available online: http://calcium.uhnres.utoronto.ca/ctdb/pub_pages/search/index.htm), and ATPint (Available online: http://www.imtech.res.in/raghava/atpint/submit.html) servers. Signal peptides were predicted using Signa1P 3.0 (Available online: http://www.cbs.dtu.dk/service/SignalP/) [60]. *N*-glycosylation sites were predicted by NetNGlyc 1.0 Server (Available online: http://www.cbs.dtu.dk/services/NetNGlyc/). DNAMAN 6.0 (Lynnon BioSoft, Vaudreuil, QC, Canada) was used to edit *TcPMCA1* nucleotide sequences, and the corresponding phylogenetic trees were constructed using the neighbor-joining method, with 1000 bootstrap replicates, in MEGA5.01 [61].

### 4.5. Real-Time Quantitative PCR (qPCR)

Primers used for qPCR were designed by Primer 3.0 software [62]. qPCR was performed in 20 µL-reaction mixture that contained 10 µL of qSYBR Green Supermix (BIO-RAD laboratories, Hercules, CA, USA), 1 µL of cDNA template, 1 µL of each primer (0.2 mM) and 7 µL of ddH_2_O. qPCR was performed on a Stratagene Mx3000P Thermal Cycler (Stratagene, La Jolla, CA, USA) as following protocol: an initial denaturation at 95 °C for 2 min, followed by 40 cycles at 95 °C for 15 s, 60 °C for 30 s, and elongation at 72 °C for 30 s. At the end of each reaction, a melt curve analys (from 60 to 95 °C) was generated to rule out the possibility of primer-dimer formation. *RPS18* was used as a stable housekeeping gene for the qPCR analysis [63]. Relative gene expression levels were calculated by 2^−ΔΔ*C*t^ method [64]. Three biological and two technical replicates were performed.

*Expression pattern of TcPMCA1 at different developmental stages.* To investigate the expression patterns of *TcPMCA1* at different developmental stages, we collected mites in uniform developmental stages (2000 eggs, 1500 larvae, 1000 nymphs, and 500 adults). The samples were isolated and placed in a 1.5 mL diethyl pyrocarbonate (DEPC)-treated centrifuge tube containing RNA storage reagent (Tiangen, Beijing, China), immediately frozen in liquid nitrogen, and stored at −80 °C for RNA extraction. Three independent biological replications were performed.

*Expression levels of TcPMCA1 after scopoletin exposure*. The differential expression levels of *TcPMCA1* in response to scopoletin were investigated by exposing adult female mites to LC_10_, LC_30_, and LC_50_ scopoletin, as in leaf bioassays. After 12, 24, and 36 h intervals, only the surviving adults obtained from the treated and control groups (at least 500 larvae) were collected and frozen at −80 °C for RNA extraction. After scopoletin exposure, total RNA was isolated to analyze the expression levels of *TcPMCA* by TR-qPCR.

### 4.6. Homology Modeling

The homology modeling was conducted on the I-TASSER server (Available online: http://zhanglab.ccmb.med.umich.edu/I-TASSER/) [65], and the 3D structure of TcPMCA1 protein was obtained. The details of I-TASSER protocol have been described previously [66,67,68,69,70]. Briefly, it consists of three steps: template identification, full-length structure assembly and structure-based function annotation. Firstly, starting from the query sequence, I-TASSER identifies homologous structure templates from the PDB library [71] using LOMETS [69,72], a meta-threading program that consists of multiple threading algorithms. Then, the topology of the full-length models is constructed by reassembling the continuously aligned fragment structures excised from the templates, where the structures of the unaligned regions are built from scratch by *ab initio* folding based on replica-exchange Monte Carlo simulations [73]. The low free-energy states are further identified by SPICKER [74]. To refine the structural models, a second round of structure reassembly is conducted starting from the SPICKER clusters. The low free-energy conformations refined by full-atomic simulations using FG-MD [75] and ModRefiner [76]. Finally, the biological functions of the target proteins were derived by matching the I-TASSER models with proteins in the BioLiP library [77,78,79].

Based on identity with the primary sequence of the target *TcPMCA1*, the crystal structure of the phosphoenzyme intermediate of the rabbit SERCA Ca^2+^-ATPase (PDB ID code: 3BA6) was retrieved from the Protein Data Bank (PDB, Available online: http://www.rcsb.org/pdb/home/home.do) and used as the template for homology modeling (the amino acid sequences of the trmplate was shown in Figure S1). The Psi/Phi Ramachandran plot obtained from Procheck analysis was used to validate the modeled 3D structure of TcPMCA1 protein [80,81].

### 4.7. Dataset and Molecular Modeling

The acaricidal activities of the 30 collected compounds (Table S3) were obtained from a previous study [82]. These 30 compounds are natural or synthetic compounds that are readily available to coumarin, which were purchased from Chengdu Aikeda Chemical Reagent Co., Ltd. and Shanghai yuanye Bio-Technology Co., Ltd. The purity of these compounds was more than 98%. The structures and half-maximal inhibitory concentration (LC_50_) of the compounds are shown in Table 2. These values were transformed into the corresponding pLC_50_ [−log(LC_50_)] as the expression of inhibitor potency. The 30 compounds were placed in a training set of 24 compounds (80%) and a test set of 6 compounds (20%).

The 3D structures of these ligand compounds were constructed in Sybyl 6.9 (Tripos Software, St. Louis, MO, USA). Structures were energy minimized by using the Gasteiger–Hückel charge [83], Tripos force field [84], and Powell methods [85] with a convergence criterion of 0.005 kcal/(mol Å). The iterations maximum number was set to 10,000, and multiple conformation search was used. Coumarin structure was used as the common scaffold for molecular alignment, and compound 2 with the highest acaricidal activity was used as the template molecule. All other compounds were aligned with the coumarin core using the “align database” command in Sybyl.

### 4.8. Molecular Docking

The protein model was prepared using Sybyl prior to docking simulations. All bound water molecules and ligands were removed from the protein, and hydrogen atoms and AM1-BCC charges [86] were added to the amino acid residues. The generated homology model of TcPMCA1 was used for molecular docking, and the binding pocket was defined using Discovery Studio 2.5 (Accelrys Software Inc., San Diego, CA, USA). The 3D structure of the compound was prepared as the ligand, and all of the hydrogen atoms and AM1-BCC charges were added [86]. Molecular docking was performed with AutoDock 4.0 [87]. The grid spacing was changed from 0.375 nm, and the cubic grid map was 40 × 40 × 40 Å toward the TcPMCA binding site. The docking parameters were set as follows: the number of GA Runs was set as 10, population size was set as 150, the maximum number of evaluations was set as 25,000,000, and 250 runs were performed. All other parameters were set as the default. The docking process was performed as follows: first, molecular docking was performed to evaluate the docking poses. Then, defined docking was conducted on the binding pocket. Three to six independent docking calculations were conducted. The corresponding lowest binding energies and predicted inhibition constants (p*K*_i_) were obtained from the docking log files (dlg). The mean ± SD of binding energies was calculated from the dockings. AutoDock Tools and Visual Molecular Dynamics (VMD, Theoretical and Computational Biophysics group at the Beckman Institute, University of Illinois at Urbana-Champaign) [88,89] was used to visualize the docking result. Surface representation images that show the binding pocket of TcPMCA1 were generated using VMD software.

### 4.9. 3D-QSAR Study

CoMFA and CoMSIA descriptor fields were employed in the present 3D-QSAR studies. The CoMFA fields were carried out to generate the steric and electrostatic fields with the default value of the energy cutoff at 30 kcal·mol^−1^ CoMSIA fields were carried out to calculate the steric, electrostatic, hydrophobic, hydrogen-bond donor and hydrogen-acceptor donor with a default attenuation factor of 0.3 for Gaussian function. Field type “Stdev * Coeff” was used as the coefficient to analysis the contour map of each field. The partial least squares (PLS) [90] was used to construct a linear correlation by setting the biological activity (pLC_50_ values) as the dependent variables and the CoMFA/CoMSIA descriptors as independent variables.

### 4.10. Statistical Analysis

All results were expressed as the mean ± standard error. The differences among the four developmental stages and time-dependent responses to scopoletin exposure were analyzed using one-way analysis of variance (ANOVA). The level of significance of the means was then separated by Fisher’s LSD multiple comparison test (*p* < 0.05). The fold change in *TcPMCA* gene expression was analyzed using SPSS (v.16.0, SPSS Inc., Chicago, IL, USA), and significance was determined by independent sample *t*-test (*p* < 0.05).

## 5. Conclusions

The molecular characteristics of the *TcPMCA1* gene were identified and described, and the gene expression levels of *TcPMCA1* after scopoletin exposure were investigated. The *TcPMCA1-*mediated detoxification mechanism of scopoletin in *T. cinnabarinus* was preliminarily explored through the integrated study of homology modeling and molecular docking. Moreover, CoMFA and CoMSIA 3D-QSAR studies have been performed to put the pharmacophoric environment that will help future structure based drug design. The results of the present study showed that scopoletin forms hydrogen bonds with the active site of *TcPMCA1*, and that the C3, C6, and C7 positions in the skeletal structure of coumarins are the most suitable active sites. These results provide a better understanding of the *TcPMCA1*-mediated detoxification mechanisms of scopoletin and of other coumarin derivatives. These compounds can be structurally modified to increase their acaricidal and inhibitory effects. More detailed investigations of the mechanism of action and pharmacological activities of these compounds may provide novel anti-PMCA agents for spider mite control.

## Figures and Tables

**Table 1:** Docking results of coumarins with Ca^2+^-ATPase 1 gene of *Tetranychus cinnabarinus* (*TcPMCA1*).

Compound	AutoDock				Compound	AutoDock			
	Einter	Eintra	Etors	∆G		Einter	Eintra	Etors	∆G
1	−6.87	−0.47	1.19	−5.64	16	−4.64	−0.15	0.3	−4.35
2	−7.22	−0.59	1.19	−6.71	17	−4.77	−0.37	0.6	−5.01
3	−4.55	−0.56	0.3	−4.65	18	−5.95	−0.86	0.89	−5.03
4	−4.95	−0.02	0.3	−5.07	19	−4.84	−1.45	0.89	−4.33
5	−4.65	−0.1	0.3	−4.38	20	−4.67	−1.13	0.6	−4.69
6	−4.95	0.03	0.3	−4.41	21	−4.61	−1.27	0.6	−4.23
7	−6.01	−0.55	0.89	−5.14	22	−4.29	0.02	0.3	−4.32
8	−4.86	−0.09	0.3	−5.04	23	−3.97	0	0	−4.47
9	−6.56	−1.73	0.89	−5.24	24	−5.89	−0.38	0.89	−6.08
10	−4.66	0.03	0.3	−3.83	25	−4.89	−0.25	0.6	−6.1
11	−4.35	−0.06	0.3	−4.59	26	−4.12	0	0	−4.59
12	−4.79	0.01	0.3	−4.58	27	−5.59	−0.59	0.89	−5.25
13	−4.56	−0.26	0.6	−4.84	28	−4.91	−0.11	0.3	−5.13
14	−4.49	0	0	−5.28	29	−4.54	−0.68	0.3	−5.35
15	−4.56	−0.14	0.6	−4.36	30	−4.42	−0.89	0.89	−4.6

1, 3-(2-benzimidazolyl)-7-(diethylamino)coumarin; 2, 3-(2-benzothiazolyl)-7-(diethylamino)coumarin; 3, 3-Aminocoumarin; 4, 3-Acetylcoumarin; 5, 4-Methoxycoumarin; 6, 4-Hydroxycoumarin; 7, 5,7-dihydroxy-4-phenyl coumarin; 8, 6-Nitrocoumarin; 9, 7,8-dihydroxy-4-phenyl coumarin; 10, 7-amino-4-phenyl coumarin; 11, 7-methoxycoumarin(herniarin); 12, 7-mercapto-4-methyl coumarin; 13, 6,7-dimethoxy coumarin(Scoparone); 14, Psoralen; 15, 7-Hydroxy-6-methoxycoumarin(Scopoletin); 16, Xanthotoxin; 17, Pimpinellin; 18, Imperatorin; 19, Fraxetin; 20, Esculetin; 21, Daphnetin; 22, Umbelliferone; 23, Coumarin; 24, Oxypeucedanin; 25, Isopimpinellin; 26, 6-Methylcoumarin; 27, Osthole; 28, Bergapten; 29, Xanthotol; 30, Isofraxidin.

**Table 2:** Structures and acaricidal activities (LC_50_ values) of the compounds tested in this study.

Compound	Structure	LC_50_ (mmol/L)	Compound	Structure	LC_50_ (mmol/L)
**1a**	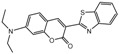	1.2175	**16a**	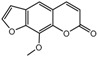	6.0313
**2a**	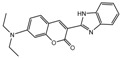	0.8638	**17a**	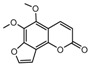	5.188
**3a**	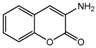	2.971	**18a**	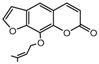	5.3789
**4a**	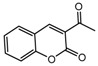	3.52	**19a**	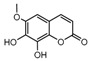	6.2036
**5b**	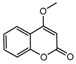	2.2563	**20a**	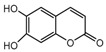	12.6973
**6b**	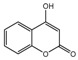	61.2926	**21b**	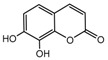	3.8273
**7a**	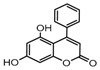	22.784	**22a**	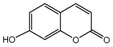	20.0142
**8a**	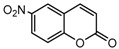	3.319	**23a**	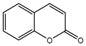	14.1447
**9b**	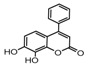	5.4987	**24a**	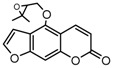	4.876
**10b**	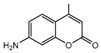	14.1318	**25a**	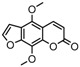	5.0816
**11a**	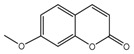	33.8571	**26a**	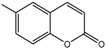	15.4398
**12b**	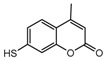	22.269	**27a**	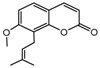	1.9186
**13a**	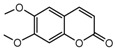	1.3813	**28a**	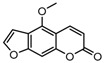	15.1358
**14a**	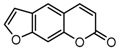	25.6564	**29a**	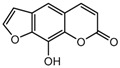	3.8
**15a**	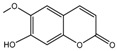	6.4698	**30a**	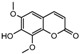	2.5798

a, Training compounds; b, test set compounds. The others are the same as those in Table 1.

**Table 3:** Summary of the results obtained from CoMFA (comparative molecular field analysis) and CoMSIA (comparative molecular similarity index analysis) analyses.

Statistical Parameter	CoMFA Model	CoMSIA Model
*q*^2^	0.750	0.710
ONC	7	6
R^2^	0.993	0.975
SEE	0.042	0.080
F	383.856	124.834
R^2^pred	0.6465	0.931
Contribution		
Steric	0.726	0.140
Electrostatic	0.274	0.334
Hydrophobic		0.239
H-bond donor		0.197
H-bond acceptor		0.090

**Table 4:** Observed and predicted activities of the test compounds.

Compound	pLC_50_	CoMFA		CoMSIA	
		Predicted pLC_50_	Residual	Predicted pLC_50_	Residual
**1a**	2.915	2.868	0.047	2.924	−0.009
**2a**	3.064	3.097	−0.033	3.021	0.043
**3a**	2.527	2.514	0.013	1.83	0.697
**4a**	2.453	2.487	−0.034	2.465	−0.012
**5b**	2.647	1.651	0.996	1.92	0.727
**6b**	1.213	2.328	−1.115	1.916	−0.703
**7a**	1.642	1.394	0.248	1.65	−0.008
**8a**	2.479	2.894	−0.415	2.493	−0.014
**9b**	2.260	1.67	0.59	1.917	0.343
**10b**	1.850	2.097	−0.247	1.857	−0.007
**11a**	1.470	2.184	−0.714	1.716	−0.246
**12b**	1.652	2.245	−0.593	1.756	−0.104
**13a**	2.860	2.258	0.602	2.779	0.081
**14a**	1.591	2.271	−0.68	1.739	−0.148
**15a**	2.189	1.84	0.349	2.18	0.009
**16a**	2.220	1.703	0.517	2.127	0.093
**17a**	2.285	1.931	0.354	2.344	−0.059
**18a**	2.269	2.304	−0.035	2.263	0.006
**19a**	2.207	2.309	−0.102	2.311	−0.104
**20a**	1.896	1.947	−0.051	1.769	0.127
**21b**	2.417	2.74	−0.323	2.063	0.354
**22a**	1.699	1.841	−0.142	1.641	0.058
**23a**	1.849	2.583	−0.734	1.806	0.043
**24a**	2.312	2.008	0.304	2.298	0.014
**25a**	2.294	1.967	0.327	2.265	0.029
**26a**	1.811	2.122	−0.311	1.765	0.046
**27a**	2.717	1.759	0.958	2.697	0.02
**28a**	1.820	1.697	0.123	1.683	0.137
**29a**	2.420	2.152	0.268	1.832	0.588
**30a**	2.588	1.681	0.907	3.694	−1.106

a, Training compounds; b, test set compounds. The others are the same as those in Table 1. CoMFA, comparative molecular field analysis; CoMSIA, comparative molecular similarity index analysis; pLC_50_, −log(LC_50_).

## Supplementary Materials

Supplementary materials can be found at www.mdpi.com/1422-0067/18/7/1380/s1.
