# Supplementary material for: 3D-QSAR and Molecular Docking Studies on the TcPMCA1-Mediated Detoxification of Scopoletin and Coumarin Derivatives
Source: Int J Mol Sci. 2017 Jun 27;18(7):1380. doi: 10.3390/ijms18071380 (PMC5535873; doi:10.3390/ijms18071380)
Supplement: Supplementary file 1 [file ijms-18-01380-s001.pdf]

Supplementary Materials:

3D-QSAR and molecular docking studies on the *TcPMCA1*-mediated detoxification of scopoletin and coumarin derivatives

Qiu-li Hou#, Jin-xiang Luo#, Bing-chuan Zhang, Gao-fei Jiang, Wei Ding, and Yong-qiang Zhang*
Laboratory of Natural Products Pesticides, College of Plant Protection, Southwest University, Chongqing, 400715, P. R. China; houqiuli2000@126.com (Q.H.); xiangxiangnx@sohu.com (J.L.); zhbichting@163.com (B.Z.); Gaofei.Jiang@toulouse.inra.fr (G.J.); dwing818@163.com (W.D.); zyqiang@swu.edu.cn (Y.Z.)
#These two authors contributed equally to this work.
* Correspondence: zyqiang@swu.edu.cn; Tel./Fax: +86-23-6825-0218

Table S1. Primer sequences used in cloning and quantitative real-time PCR

Experiments	Primer names and sequences (5' to 3')	Product length (bp)
Specific PCR	TcPMCA1-F: CGGGATCCCCTGCCCTTTGACATTTA<br>TcPMCA1-R: CCGCTCGAGCACGGACTTCATACCTCG	2000
5' RACE	TcPMCA1-F1: GTTTTAGGTGGTTTAGGAGGA<br>TcPMCA1-F2: TCCTCTAAATCTTTGGGTGA	667
3' RACE	TcPMCA1-R1: TCGAAACAGAGCCAAATTGGGAAGA<br>TcPMCA1-R2: ATACTTGGCATCGAAGATCCCGTCC	1355
Full-length confirmation	TcPMCA1-1: CGGGATCCCCTGCCCTTTGACATTTA<br>TcPMCA1-2: CGCTCGAGCACGGACTTCATACCTCG	2027
	TcPMCA1-3: CGGGATCCCACGGACTTCATACCTCG<br>TcPMCA1-4: CCGCTCGAGCACCAAAGCCATTGTTCT	3056
	TcPMCA1-5: CGGGATCCCACGGACTTCATACCTCG<br>TcPMCA1-6: CCGCTCGAGGCACCCAATAAAGCAAAT	683
RT-qPCR	TcPMCA1-Q-F: GTTTGGCCTCGTTTACGTGT<br>TcPMCA1-Q-R: TTCCTTGGCTACATCGGTTC	201
RPS18	RPS18-F: ACGTGCTGGTGAACTTACCGAAGA<br>RPS18-R: TGCCTATTCAAGAACCAAAGTGGG	99

Table S2. Sequences and relevant information used in the phylogenetic analysis of plasma membrane Ca^{2+}-ATPase (PMCA).

Organism	Accession number
**Ixodes**	
Tetranychus urticae PMCA1	tetur02g05600
Tetranychus urticae PMCA2	tetur09g02350
Tetranychus urticae SERCA1	tetur18g02100
Ixodes scapularis PMCA2	EEC15444.1
Ixodes scapularis E1-E2 ATPase	EEC11414.1
**Cnidaria (Outgroup)**	
Stylophora pistillata	AY360080
**Insect**	
Aedes aegypti	XP_001651107.1
Anopheles gambiae	XP_311357.4
Apis mellifera PMCA3	XP_003250350.1
Acythosiphon pisum PMCA1	XP_001945158.1
Acythosiphon pisum PMCA2	XP_001950494.1
Camponotus floridanus	EFN65040.1
Culex quinquefasciatus	XP_001847165.1
Drosophila melanogaster	NP_001033803.3
Drosophila mojavensis	XP_002011413.1
Danaus plexippus	EHJ74187.1
Pediculus humanus corporis	XP_002427234.1
Tribolium castaneum	XP_966434.1
**Chordate**	
Branchiostoma floridae	XP_002586028.1
Ciona intestinalis	XP_002126083.2
Danio rerio PMCA1	NP_001038222.1
Danio rerio PMCA2	NP_001116710.1
Danio rerio PMCA3	NP_001002472.1
Danio rerio PMCA4	EU559285.1
Gallus gallus PMCA1	NP_001161474.1
Gallus gallus PMCA2	XP_001231642.1
Gallus gallus PMCA4	XP_418055.2
Homo sapiens PMCA1	NP_001001323.1
Homo sapiens PMCA2	NP_001001331.1
Homo sapiens PMCA3	NP_068768.2
Homo sapiens PMCA4	NP_001001396.1
Mus musculus PMCA1	NP_080758.1
Mus musculus PMCA2	NP_033853.1
Mus musculus PMCA3	NP_796210.2
Mus musculus PMCA4	NP_998781.1
Ornithorhynchus anatinus PMCA1	XP_001512183.1
Ornithorhynchus anatinus PMCA3	XP_001510240.1
Ornithorhynchus anatinus PMCA4	XP_001518636.1
Xenopus laevis PMCA1	EU752492.1
Xenopus laevis PMCA2	NP_001087020.1
Xenopus (Silurana) tropicalis PMCA3	NP_001124515.1
**Nematoda**	
Caenorhabditis briggsae PMCA1	XP_002633689.1
Caenorhabditis briggsae PMCA2	XP_002634908.1
Caenorhabditis briggsae PMCA3	XP_002633968.1
Caenorhabditis elegans PMCA1	NP_741352.1
Caenorhabditis elegans PMCA2	NP_500161.1
Caenorhabditis elegans PMCA3	NP_501709.2

Table S3. Acaricidal activities of test compounds

Compound	LC_{50}				R	Compound.	LC_{50}				R
	(mg/L)		(mmol/L)				(mg/L)		(mmol/L)		
	LC_{50}	SE(95% CL)	LC_{50}	SE(95% CL)			LC_{50}	SE(95% CL)	LC_{50}	SE(95% CL)	
**1a**	0.4059	0.0493	1.2175	0.1479	0.9929	**16a**	1.3039	0.2671	6.0313	1.2355	0.9947
**2a**	0.3027	0.0234	0.8638	0.0668	0.9933	**17a**	1.2774	0.2075	5.188	0.8427	0.9612
**3a**	0.4788	0.0338	2.971	0.2097	0.9979	**18a**	1.4538	0.2574	5.3789	0.9523	0.9857
**4a**	0.6624	0.0696	3.52	0.3699	0.967	**19a**	1.2914	0.2317	6.2036	1.113	0.9958
**5b**	0.3975	0.0524	2.2563	0.2974	0.976	**20a**	2.2619	0.7932	12.6973	4.4527	0.8523
**6b**	9.9386	1.0688	61.2926	6.5914	0.9283	**21b**	0.6818	0.0878	3.8273	0.4929	0.9838
**7a**	5.7926	2.5973	22.784	10.2159	0.993	**22a**	3.2451	1.1658	20.0142	7.1901	0.955
**8a**	0.6344	0.1312	3.319	0.6864	0.8435	**23a**	2.0671	0.3547	14.1447	2.4271	0.9243
**9b**	1.398	0.3235	5.4987	1.2724	0.9775	**24a**	1.3959	0.313	4.876	1.0933	0.9932
**10b**	2.4756	0.5829	14.1318	3.3274	0.9903	**25a**	1.2512	0.155	5.0816	0.6295	0.9972
**11a**	5.9646	0.9378	33.8571	5.3233	0.9206	**26a**	2.473	0.7258	15.4398	4.5314	0.9922
**12b**	4.281	0.905	22.269	4.7077	0.9906	**27a**	0.4687	0.0504	1.9186	0.2063	0.9697
**13a**	0.2848	0.0302	1.3813	0.1465	0.9824	**28a**	3.2722	1.4539	15.1358	6.7251	0.9782
**14a**	4.7762	0.6985	25.6564	3.7521	0.9951	**29a**	0.7682	0.1033	3.8	0.511	0.9532
**15a**	1.2433	0.1278	6.4698	0.665	0.9618	**30a**	0.5732	0.0526	2.5798	0.2367	0.9139

R: Relative coefficient.
a: training compounds; **b:** test set compounds; the others same to **Table 2**.

1 ATGGAGGCCGCGCACTCCAAGAGCACAGAGGAATGTCTGGCCTATTTTGGGGTGAGCGAGACCACAGGCCTCACCCCGGACCAAGTGAAG
1 M E A A H S K S T E E C L A Y F G V S E T T G L T P D Q V K

91 CGACATCTAGAGAAATACGGCCACAATGAGCTTCCTGCTGAGGAAGGGAAATCCCTGTGGGAGCTGGTGATAGAGCAGTTTGAAGACCTC
31 R H L E K Y G H N E L P A E E G K S L W E L V I E Q F E D L

181 CTGGTGCGGATTCTTCTGCTGGCCGCCTGCATCTCCTTTGTGCTGGCCTGGTTTGAAGAAGGGGAAGAGACCATCACTGCCTTCGTTGAG
61 L V R I L L L A A C I S F V L A W F E E G E E T I T A F V E

271 CCCTTTGTCATCCTCCTGATCCTCATTGCCAATGCCATCGTGGGAGTTTGGCAGGAGCGGAACGCTGAGAACGCCATAGAGGCGCTGAAG
91 P F V I L L I L I A N A I V G V W Q E R N A E N A I E A L K

361 GAATATGAGCCCGAGATGGGGAAGGTGTACCGGGCTGACCGCAAGTCAGTGCAAAGGATCAAGGCTCGGGACATCGTCCCCGGGGACATC
121 E Y E P E M G K V Y R A D R K S V Q R I K A R D I V P G D I

451 GTGGAGGTGGCGGTTGGGGACAAAGTCCCTGCAGACATCCGCATCCTGTCTATCAAGTCCACCACCCTCCGCGTGGACCAGTCCATCCTG
151 V E V A V G D K V P A D I R I L S I K S T T L R V D Q S I L

541 ACAGGCGAGTCTGTGTCCGTCATCAAGCACACGGAGCCAGTCCCTGACCCGCGGGCTGTCAACCAGGACAAGAAGAACATGCTTTTCTCG
181 T G E S V S V I K H T E P V P D P R A V N Q D K K N M L F S

631 GGTACCAACATCGCGGCCGGCAAGGCCCTGGGCATCGTGGCCACCACCGGCGTGAGCACCGAGATCGGGAAGATCCGTGACCAGATGGCC
211 G T N I A A G K A L G I V A T T G V S T E I G K I R D Q M A

721 GCCACGGAGCAGGACAAGACGCCGCTGCAGCAGAAGCTGGATGAGTTCGGGGAGCAGCTGTCCAAGGTCATCTCCCTCATCTGCGTGGCC
241 A T E Q D K T P L Q Q K L D E F G E Q L S K V I S L I C V A

811 GTGTGGCTTATCAACATCGGCCACTTCAACGACCCCGTCCACGGGGGCTCCTGGATCCGCGGTGCCATCTACTACTTCAAGATCGCCGTG
271 V W L I N I G H F N D P V H G G S W I R G A I Y Y F K I A V

901 GCCTTGGCTGTGGCTGCGATCCCAGAAGGTCTTCCCGCTGTCATCACTACCTGCCTGGCCCTGGGCACCCGCCGGATGGCGAAGAAGAAC
301 A L A V A A I P E G L P A V I T T C L A L G T R R M A K K N

991 GCCATCGTGAGGAGCCTGCCCTCTGTGGAGACCCTGGGCTGCACCTCTGTCATCTGCTCTGACAAGACTGGCACCCTCACCACCAACCAG
331 A I V R S L P S V E T L G C T S V I C S D K T G T L T T N Q

1081 ATGTCTGTGTGCAAGATGTTCATCATCGACAAGGTGGACGGAGACTTCTGTTCGCTGAACGAGTTCTCCATCACCGGCTCCACCTACGCT
361 M S V C K M F I I D K V D G D F C S L N E F S I T G S T Y A

1171 CCAGAGGGGGAGGTCCTGAAGAATGATAAACCCATCCGGTCAGGGCAGTTTGATGGGCTGGTGGAGCTGGCCACCATTTGTGCCCTGTGC
391 P E G E V L K N D K P I R S G Q F D G L V E L A T I C A L C

1261 AATGATTCCTCCTTGGACTTCAATGAGACCAAAGGCGTCTATGAGAAGGTGGGTGAGGCCACGGAGACGGCGCTCACCACCCTGGTGGAG
421 N D S S L D F N E T K G V Y E K V G E A T E T A L T T L V E

1351 AAGATGAATGTGTTCAACACGGAAGTTCGGAACCTCTCGAAGGTGGAGAGAGCCAACGCCTGCAACTCGGTGATCCGCCAGCTCATGAAG
451 K M N V F N T E V R N L S K V E R A N A C N S V I R Q L M K

1441 AAAGAGTTCACCCTGGAGTTCTCCCGAGACAGGAAGTCCATGTCTGTGTACTGTTCTCCAGCCAAATCTTCCCGCGCTGCTGTGGGCAAC
481 K E F T L E F S R D R K S M S V Y C S P A K S S R A A V G N

1531 AAGATGTTTGTCAAGGGCGCCCCCGAGGGGGTCATCGACCGCTGTAACTACGTGCGAGTCGGCACCACCCGGGTGCCCATGACTGGGCCG
511 K M F V K G A P E G V I D R C N Y V R V G T T R V P M T G P

1621 GTGAAGGAGAAGATCCTCTCCGTGATCAAGGAGTGGGGCACCGGCCGGGACACGCTGCGCTGCCTGGCCCTGGCCACCCGCGACACGCCG
541 V K E K I L S V I K E W G T G R D T L R C L A L A T R D T P

1711 CCCAAGCGAGAGGAAATGGTGCTGGACGACTCCTCCCGGTTCATGGAGTACGAGACGGACCTGACGTTCGTGGGCGTCGTGGGCATGCTG
571 P K R E E M V L D D S S R F M E Y E T D L T F V G V V G M L

1801 GACCCGCCCCGCAAGGAGGTCATGGGCTCCATCCAGCTGTGCCGGGACGCCGGGATCCGTGTCATCATGATCACCGGCGACAACAAGGGC
601 D P P R K E V M G S I Q L C R D A G I R V I M I T G D N K G

1891 ACGGCCATCGCCATCTGCCGCCGCATCGGCATCTTTGGGGAGAACGAGGAGGTGGCAGACCGCGCCTACACCGGCCGCGAGTTTGACGAC
631 T A I A I C R R I G I F G E N E E V A D R A Y T G R E F D D

1981 CTGCCCCTGGCCGAGCAGCGGGAAGCCTGCCGCCGCGCCTGCTGCTTCGCGCGCGTGGAACCCTCCCACAAGTCCAAGATCGTGGAATAC
661 L P L A E Q R E A C R R A C C F A R V E P S H K S K I V E Y

2071 CTGCAGTCCTACGATGAGATCACGGCCATGACAGGGGATGGCGTCAACGATGCCCCTGCCCTGAAGAAGGCCGAGATCGGCATAGCTATG
691 L Q S Y D E I T A M T G D G V N D A P A L K K A E I G I A M

2161 GGATCTGGCACCGCCGTGGCCAAGACAGCGTCTGAGATGGTCCTGGCGGACGACAACTTCTCCACCATCGTGGCCGCCGTGGAGGAGGGC
721 G S G T A V A K T A S E M V L A D D N F S T I V A A V E E G

2251 CGGGCCATCTACAACAACATGAAGCAGTTCATCCGCTACCTCATCTCCTCCAACGTGGGCGAGGTGGTCTGCATCTTCCTGACGGCCGCC
751 R A I Y N N M K Q F I R Y L I S S N V G E V V C I F L T A A

2341 TTGGGGCTGCCCGAGGCCCTGATCCCCGTGCAGCTGCTGTGGGTGAACCTGGTGACGGACGGGCTCCCGGCCACAGCCCTGGGCTTCAAC
781 L G L P E A L I P V Q L L W V N L V T D G L P A T A L G F N

2431 CCACCAGACCTGGACATCATGGACCGGCCCCCCCGGAGTCCCAAGGAGCCCCTGATCAGTGGCTGGCTCTTCTTCCGCTACATGGCCATC
811 P P D L D I M D R P P R S P K E P L I S G W L F F R Y M A I

2521 GGGGGCTATGTGGGTGCAGCCACCGTGGGAGCCGCTGCCTGGTGGTTCATGTATGCGGAGGATGGGCCGGGTGTCACCTACCACCAGCTG
841 G G Y V G A A T V G A A A W W F M Y A E D G P G V T Y H Q L

2611 ACCCACTTCATGCAGTGCACCGAGGACCACCCTCACTTTGAGGGTCTGGACTGTGAGATCTTTGAGGCCCCAGAGCCCATGACCATGGCC

871 T H F M Q C T E D H P H F E G L D C E I F E A P E P M T M A

2701 TTGTCTGTGCTGGTGACCATCGAGATGTGCAATGCTCTCAACAGCCTGTCCGAGAACCAGTCCTTGATGCGGATGCCGCCCTGGGTGAAC
901 L S V L V T I E M C N A L N S L S E N Q S L M R M P P W V N

2791 ATCTGGCTGCTGGGCTCCATCTGCCTGTCCATGTCCCTCCACTTCCTCATCCTCTACGTCGACCCCCTGCCGATGATCTTCAAGCTCAAG
931 I W L L G S I C L S M S L H F L I L Y V D P L P M I F K L K

2881 GCCCTAGACCTCACCCAGTGGCTCATGGTCCTCAAGATCTCACTTCCAGTCATCGGGCTGGATGAAATACTCAAGTTCATTGCTCGGAAC
961 A L D L T Q W L M V L K I S L P V I G L D E I L K F I A R N

2971 TACCTGGAGGATCCAGAAGATGAAAGGAGGAAGTAA
991 Y L E D P E D E R R K *

Figure S1. Nucleotide and amino acid sequence of the crystallographic structure of SERCA Ca^{2+}-ATPase used as template for homology modeling (PDB ID code: 3BA6).
